# Vietnamese chickens: a gate towards Asian genetic diversity

**DOI:** 10.1186/1471-2156-11-53

**Published:** 2010-06-18

**Authors:** C Berthouly-Salazar, X Rognon, T Nhu Van, M Gély, C Vu Chi, M Tixier-Boichard, B Bed'Hom, N Bruneau, E Verrier, JC Maillard, JR Michaux

**Affiliations:** 1 CIRAD, UPR AGIRS, Campus International de Baillarguet, F-34398 Montpellier, France; 2 AgroParisTech, UMR1313 Génétique animale et biologie intégrative F-75005 Paris, France; 3 INRA, UMR1313 Génétique animale et biologie intégrative, F-78350 Jouy-en-Josas, France; 4 NIAH, Tu Liem, Ha Noi, Vietnam; 5 Institut de Botanique (Bat. 22), University of Liège, 4000, Liège, Belgium; 6 Centre de Biologie et de Gestion des Populations (CBGP), UMR 1062, Campus International de Baillarguet, CS 30016, F-34988, Montferrier le Lez, France

## Abstract

**Background:**

Chickens represent an important animal genetic resource and the conservation of local breeds is an issue for the preservation of this resource. The genetic diversity of a breed is mainly evaluated through its nuclear diversity. However, nuclear genetic diversity does not provide the same information as mitochondrial genetic diversity. For the species *Gallus gallus*, at least 8 maternal lineages have been identified. While breeds distributed westward from the Indian subcontinent usually share haplotypes from 1 to 2 haplogroups, Southeast Asian breeds exhibit all the haplogroups. The Vietnamese Ha Giang (HG) chicken has been shown to exhibit a very high nuclear diversity but also important rates of admixture with wild relatives. Its geographical position, within one of the chicken domestication centres ranging from Thailand to the Chinese Yunnan province, increases the probability of observing a very high genetic diversity for maternal lineages, and in a way, improving our understanding of the chicken domestication process.

**Results:**

A total of 106 sequences from Vietnamese HG chickens were first compared to the sequences of published Chinese breeds. The 25 haplotypes observed in the Vietnamese HG population belonged to six previously published haplogroups which are: A, B, C, D, F and G. On average, breeds from the Chinese Yunnan province carried haplotypes from 4.3 haplogroups. For the HG population, haplogroup diversity is found at both the province and the village level (0.69).

The AMOVA results show that genetic diversity occurred within the breeds rather than between breeds or provinces. Regarding the global structure of the mtDNA diversity per population, a characteristic of the HG population was the occurrence of similar pattern distribution as compared to *G. gallus spadiceus*. However, there was no geographical evidence of gene flow between wild and domestic populations as observed when microsatellites were used.

**Conclusions:**

In contrast to other chicken populations, the HG chicken population showed very high genetic diversity at both the nuclear and mitochondrial levels. Due to its past and recent history, this population accumulates a specific and rich gene pool highlighting its interest and the need for conservation.

## Background

Chickens represent an important protein source for humans, as shown by a strong increase of poultry production around the world (USDA Foreign Agricultural Service - November 2005). Local populations contribute more specifically, to family poultry production, which is quite important for low income farmers from Africa, Asia, Latin America and the South Pacific. These local populations that are easy to raise, are resilient to harsh environmental conditions and may harbour original features of disease resistance [1]. Within this framework, chicken genetic resources have been well investigated during the last decade [2-5] using microsatellite data. Surprisingly, the number and location of chicken domestication centres are not completely clarified. The first genetic study on mtDNA suggested that the Indochinese Red Junglefowl subspecies *Gallus gallus gallus *is the primary maternal ancestor of the domestic chicken (*Gallus gallus domesticus*; [6]). Liu *et al*. [7] showed that at least three subspecies of *G. gallus *were enrolled in the origin of domestic chicken breeds, but also that there may be at least two domestication centres: one in Southeast Asia (South China, Vietnam, Myanmar and Thailand) and one in the Indian subcontinent. Furthermore, in a recent study, Errikson *et al*. [8] highlighted the hybrid origin of the domestic chicken, due to ancestral hybridisation involving the Grey Junglefowl (*G. sonneratii*).

Liu *et al*. [7] performed the widest study, involving 900 wild and domestic chickens, and found a total of 9 haplogroups to which we will always refer in this study. According to this classification, 6 to 9 haplogroups have been observed in Southeast and East Asia [7,9,10]. Some of these are localised in specific regions. For example, haplogroup C was mainly found in Japanese breeds while haplogroups G and F were found in chickens from the Chinese Yunnan province. In European, Middle-Eastern and Indian chicken breeds, 3 main haplogroups (A, B and E) have been observed [7,11]. In Latin-America, and more precisely Chile, chickens also carry the A, B and E haplogroups. The route for introduction of haplogroups A and B is still open to discussion [12,13]. In African chickens, only two haplogroups, D and E, were found [14]. The heterogeneous distribution of mitochondrial lineages implies that breeds will not carry the same genetic diversity from a maternal gene pool point of view.

On the basis of microsatellite information, African and Asian local chicken populations showed high genetic diversity [3,5,4,11]. It is interesting to note that by conserving such local breeds an important part of the gene pool is assumed to be conserved according to microsatellite data. However depending on the commercial exchange, human migration history and geographic area, just a few or on the contrary almost all the maternal lineages will be conserved. Therefore, it is important to have a global view (i.e. mtDNA and microsatellites) of the genetic make-up of chicken breeds as much as possible.

In a previous analysis, we demonstrated that the local population of Vietnamese chickens, namely the H'mong chicken, showed a high genetic diversity and could not be subdivided into subpopulations [15]. This population showed almost all possible phenotypes such as silky feathers as well as frizzled plumage. The only phenotype not observed was the naked neck (Na gene) (personal observation). In addition, we highlighted that admixture may occur between wild and domestic chickens in a few localised communes. In this paper, we present a first assessment of mtDNA polymorphism in this local population. We generated mtDNA control region sequences from 106 H'mong chickens and our wild Red junglefowl samples and compared the results with those already published for the wild Red junglefowl and Asian chicken breeds. Three major questions may be addressed: (1) is the mtDNA genetic diversity as high as the diversity observed at the nuclear level; (2) how has geographical isolation affected the distribution of mtDNA haplotypes and (3) are there occurrences of admixture between wild and domestic chickens?

## Methods

### Sample collection

Vietnamese chickens originate from the northern Ha Giang province (22°08' - 23°19' N; 104°33' - 105°33' E) bordering the Yunnan Chinese province. The local chicken population from this province was previously considered to belong to the H'mong Black skin chicken. However previous genetic data demonstrated that no breed differentiation and no congruence between the black phenotype and the genetic structure occurred in the province [15]. Therefore, instead of naming this population as the H'mong chicken, which for Vietnamese people indicates only animals with the black skin phenotype, we will refer to it as the Ha Giang (HG) chicken population. According to Berthouly *et al*. [15], chickens originating from four communes seemed to be admixed with wild junglefowls. We sequenced 68 chickens from these four admixed communes and 38 chickens from six non-admixed communes. Thus, a total of 106 mtDNA D-loop sequences from 40 villages among 10 communes were analysed (Table 1). We also sequenced the 3 wild populations previously studied in Berthouly *et al*. [15]: two populations of *G. g. gallus *(Gg2 from Thailand, and Gg3 from Vietnam) and one population of *G. g. spadiceus *from Thailand (Gg1). Thus, a total of 30 new sequences of Red junglefowl were added.

**Table 1:** List of Vietnamese communes sampled, diversity measures and geographical distribution of haplogroups within the Ha-Giang province: the number of samples *Ni*, the haplotype diversity *h*, the nucleotide diversity *π*

	*Ni*	Nb haplotypes	Nb haplo- groups	Haplogroups		*h*	π	
Non admixed communes								

103	8	6	3	ABG		0.86	0.010	
1	6	5	4	ACFG		0.93	0.019	
20	7	5	2	AF		0.90	0.014	
2	4	4	2	AB		1.00	0.007	
48	8	4	3	ABF		0.75	0.010	
61	5	4	3	ABD		0.90	0.014	

Admixed communes								

40	24	11	5	ABCDF		0.84	0.012	
65	9	4	2	AB		0.75	0.008	
7	6	6	4	ABFG		1.00	0.018	
88	29	11	5	ABCFG		0.86	0.011	

			Haplogroup (%)					
			
			A	B	C	D	F	G

Non admixed communes	38	25	31.6	44.7	2.6	2.6	13.2	7.9
Admixed communes	68	25	16.2	58.8	5.9	1.5	13.2	4.4

### mtDNA amplification and sequencing

Details on chicken blood collection and DNA extraction were described previously in Berthouly *et al*. [15]. Laws and regulations regarding the use of animals in scientific research have been followed. The HVS-I sequence was amplified using the same primers and PCR conditions as in Liu *et al*. [7]. PCR products were sent to MACROGEN (Macrogen Inc., Seoul, Korea) and Eurofins MGW (Germany) for sequencing using both forward and reverse strands.

### Sequence alignment

Vietnamese samples were compared with 93 wild *Gallus gallus, involving 4 subspecies *(*G. g. gallus*, *G. g. spadiceus*, *G. g. jabouillei *and *G. g.bankiva*) and 437 *Gallus gallus domesticus *(40 Chinese breeds) already analysed by Liu *et al*. [7]; and downloaded from GenBank database (Additional file 1). The sequences were aligned using BioEdit [16]. The sequences produced during this study were deposited into GenBank (Accession numbers: HM462082-HM462217).

### Analyses

#### Genetic diversity

The position and number of variable sites as well as the definition of haplotypes were computed with DnaSP 4.9 [17]. Haplotypes found in our samples were compared to haplotypes from Liu *et al*. [7] and renamed similarly when previously described. New haplotypes specific to the Vietnamese HG population were coded with the haplogroup name they belong to followed by a "V" (i.e. AV, BV, etc). Haplotype (*h*) diversity, nucleotide (*π*) diversity, pairwise differences, and a Minimum Spanning Tree (MST) from Kimura-2 parameter distances (*K2P*, [18]) between haplotypes were computed using ARLEQUIN 3.1 software [19].

#### Breed differentiation

In order to investigate the genetic differentiation among the Vietnamese and Chinese breeds (this study and [7]), analysis of molecular variance (AMOVA) and pairwise Kimura-2 parameter distances [18] between breeds were computed using the ARLEQUIN 3.1 software [19]. To visualise the paired genetic distances as plots in a low-dimensional space, multidimensional scaling analysis of these distance matrices was performed using the statistical program R [20].

#### Comparison with other published data from Asian and Indian breeds

In order to have a more general view of the distribution of chicken haplogroups, we combined our data with haplotypes published by Oka *et al*. [9] on Japanese chickens; by Kanginakudru *et al*. [11] on Indian chickens and *G. gallus murghi*, with sequences from Sri Lanka chickens published by Silva *et al*. [10] and with haplotypes described by Liu *et al*. [7]. We used version 3.7 of the MODELTEST program ([21]) to estimate which nucleotide substitution model best fitted the observed data according to the Akaike information criterion (AIC) [22]. Applying the model suggested, we performed a Maximum-likelihood (ML) analysis with PhyML [23] and the starting tree was determined by a BioNJ analysis of the data sets (default settings). One thousand bootstrap replicates were performed using optimisation options,.

#### Results & Discussion

**Figure 1:**
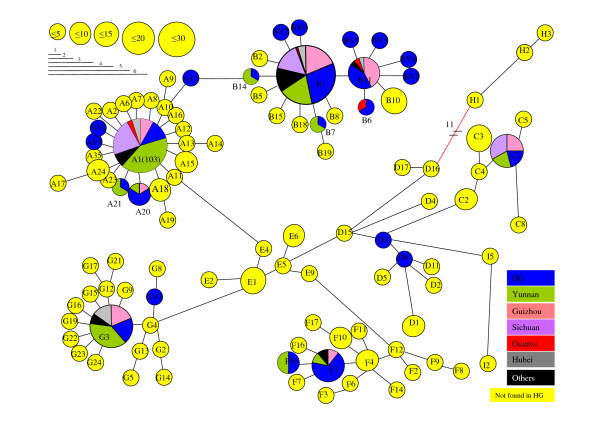
**Minimum spanning tree of the Vietnamese mtDNA haplotypes combined with the Chinese mtDNA from Liu *et al*. **[7]. Circle size corresponds to haplotype frequencies. Haplotypes that are not found in the HG populations are in yellow, those that have been observed in wild populations are written in red. When haplotype is found both in the HG population, and in Chinese provinces, proportions are indicated

For the HG chicken population, we obtained a segment of 506 bp of mtDNA HVS-I sequence. Among the 106 sequences, 33 variables sites were found, involving 36 mutational events, which defined 25 haplotypes. All but 6 mutations were transitions. The 25 haplotypes observed belonged to six haplogroups previously found by Liu *et al*. [7] which are: A, B, C, D, F and G (Figure 1). Three haplogroups were not encountered in the HG chicken population, namely I, which has only been observed in three *Gallus gallus *from Hanoï, H (Indonesia, wild junglefowls with unknown origin, and sequences from the Indian subcontinent) and E (Indian subcontinent, Europe, Latin America and Africa) [7,11-13]. The absence of clade E suggests that this Vietnamese population has not yet been admixed with Indian breeds but most of all it was not admixed with highly productive European breeds.

From our *G. g. gallus *Gg3 population from Vietnam, 2 haplotypes, G6 and G25 were observed. This corroborates the result from Liu *et al*. [7], who only observed G6 haplotype in his Vietnamese wild sample. We, found for the first time, haplotypes belonging to clade A (i.e. A1) in a *G. gallus gallus *population (i.e. Gg2). Haplotypes from clade A were not observed in such a population in previous published studies. In both populations from Thailand, Gg1 and Gg2, we found three new haplotypes from clade I. Until now, this clade was only represented by 2 haplotypes in samples originating from Vietnam [7]. Thus, clade I has been also evolving in Thailand and not only in Vietnam.

### Genetic diversity within and between breeds from Vietnam and China

In the HG population, haplotype diversity *(h) *and nucleotide diversity *(π) *per commune ranged from 0.75 to 1 (mean *h *= 0.86 ± 0.088) and from 0.007 to 0.019 (mean *π *= 0.013 ± 0.004) respectively (Table 1), indicating that this genetic diversity is distributed across the province. Haplotype diversity and nucleotide diversity of Chinese breeds ranged from 0 to 1 and from 0 to 0.019 respectively (Table 2). The highest nucleotide diversity was found in the Chinese Yunnan province (mean *π *= 0.013 ± 0.005).

**Table 2:** List of breeds, diversity measures and geographical distribution of haplogroups: the number of samples *Ni*, the haplotype diversity *h*, the nucleotide diversity *π*.

Breed names	Code	Province	Country	*Ni*	Number of haplotypes	*h*	π	Haplogroups (%)							Nb of haplogroups
									
								A	B	C	D	E	F	G	
Beijing Youkei	BY	Beijing	China	4	3	0.83	0.006	75	25	0	0	0	0	0	2

Huxu	HX	Guangdong	China	5	1	0.00	0.000	0	0	100	0	0	0	0	1

Qinyuan blotted	QY	Guangdong	China	10	3	0.60	0.011	0	33	67	0	0	0	0	2

Luke	LK	Guanxi	China	9	4	0.81	0.013	44	22	33	0	0	0	0	3

Wanfeng Wugu	Wangf	Guanxi	China	9	5	0.81	0.016	44	11	11	0	0	11	22	5

Dwarf Wugu	Dwarf	Guizhou	China	10	5	0.86	0.012	27	27	0	0	45	0	0	3

Guizhou Moutain Wugu	Wguiz	Guizhou	China	10	5	0.76	0.012	64	0	18	0	27	0	0	3

Heikang Layer	HK	Guizhou	China	5	2	0.60	0.013	60	0	40	0	0	0	0	2

Wumeng Wugu	WM	Guizhou	China	4	2	0.67	0.003	0	100	0	0	0	0	0	1

Gushi Wugu	Wgus	Henan	China	11	6	0.89	0.012	42	8	0	0	42	0	8	4

Henan Cockfight	DJ	Henan	China	4	1	0.00	0.000	25	58	0	0	17	0	0	3

Taihe Silky	SY	Henan	China	14	3	0.65	0.010	36	50	14	0	0	0	0	3

Black Silky hubei	HBHF	Hubei	China	10	3	0.60	0.005	40	60	0	0	0	0	0	2

Silky hybrid	KD	Hubei	China	3	2	0.67	0.009	33	67	0	0	0	0	0	2

Silky hubei	HBBF	Hubei	China	11	3	0.69	0.011	36	45	18	0	0	0	0	3

Yunxian Wugu	Y	Hubei	China	10	5	0.87	0.014	20	30	50	0	0	0	0	3

Xuefeng	XF	Hunan	China	8	4	0.64	0.008	0	75	13	0	13	0	0	3

Langshan	RS	Jiangsu	China	4	1	0.00	0.000	0	100	0	0	0	0	0	1

Silky Jiangsu	JSW	Jiangsu	China	13	6	0.85	0.013	38	23	31	0	0	8	0	4

Yugan wugu	YG	Jiangxi	China	4	4	1.00	0.015	25	25	0	0	50	0	0	3

Bigbone	DG	Liaoning	China	4	2	0.50	0.005	25	75	0	0	0	0	0	2

Souguang	SG	Shangdong	China	4	3	0.83	0.007	25	75	0	0	0	0	0	2

Caoke	Caoke	Sichuan	China	16	6	0.62	0.004	94	0	0	0	6	0	0	2

Chengdu Black Silky	ChenBlaSil	Sichuan	China	9	5	0.81	0.010	44	44	0	0	0	0	11	3

Chengdu Silky	ChenSil	Sichuan	China	6	4	0.71	0.004	100	0	0	0	0	0	0	1

SichuanMountain Wugu	sdw	Sichuan	China	11	6	0.87	0.012	25	8	25	0	42	0	0	4

Ya'an Non Wugu	YH	Sichuan	China	7	3	0.67	0.002	100	0	0	0	0	0	0	1

Ya'an Wugu	YW	Sichuan	China	10	8	0.87	0.010	44	22	11	0	22	0	0	4

Tibetan	ZJ	Tibetan	China	4	1	0.00	0.000	100	0	0	0	0	0	0	1

Chahua	Chahua	Yunnan	China	16	8	0.87	0.008	42	56	0	0	2	0	0	3

Chigulu	Chig	Yunnan	China	19	7	0.78	0.015	27	33	0	9	0	9	21	5

Douji	Douji	Yunnan	China	9	5	0.72	0.010	11	72	0	0	6	6	6	5

Jiangbian	Jiang	Yunnan	China	28	11	0.85	0.013	14	0	0	0	4	43	39	4

Lv'erwu	Lerv	Yunnan	China	29	13	0.92	0.017	24	14	3	0	10	17	31	6

Nixi	NX	Yunnan	China	9	8	0.97	0.015	9	7	0	0	2	32	50	5

Shenggou	Sheng	Yunnan	China	3	2	0.67	0.019	50	0	0	0	0	50	0	2

Tenchongxue	Teng	Yunnan	China	45	15	0.88	0.016	32	7	0	0	5	50	7	5

Whenshanshandi	When	Yunnan	China	35	12	0.89	0.015	31	28	3	0	0	14	25	5

Wuding	WD	Yunnan	China	7	4	0.81	0.010	29	57	0	0	0	0	14	3

Yanjing Wugu	YJ	Yunnan	China	8	7	0.96	0.015	13	13	13	0	0	0	63	4

HG population	HG	Ha Giang	Vietnam	106	25	0.86	0.013	21	53	5	2	0	13	6	6

*G. g. jabouillei*				3	3			33	0	0	33	0	33	0	3

*G. g. spadiceus*	(Genbank + Gg1)			47	13			15	38	0	23	0	11	0	5*

*G. g. gallus*	(Genbank + Gg2 + Gg3)			46	19			4	26	0	22	2	11	15	8*

* G. g. gallus also had haplotypes from cluster I and H that are not represented in this table

As aforementioned, six haplogroups where found in the HG province. Only the Chinese Lv'erwu breed exhibited a similar rate. On average, breeds from the Yunnan province carried haplotypes from 4.3 haplogroups. With the HG population, this haplogroup diversity was not only found at the province level, but also at the village level. For villages where at least three animals were sampled, haplotype diversity averaged 0.86 and haplogroup diversity 0.69. This means that within a village, nearly two chickens out of three carried sequences from two different matriarchal haplogroups. This is extremely high and implies that conserving chickens from only one village from the HG province would make it possible to maintain more matriarchal lineages than would the conservation of African, Chilean or Indian local chickens [11,13,14]. The HG population showed a high nuclear genetic diversity with (He > 0.60) as compared to the 11 other domestic breeds studied in Berthouly *et al*. [15]. This population exhibited the highest allelic richness (mean A = 2.9) and harboured 33 of the 36 private alleles found across all studied populations [15]. Pooling all these results together, the HG population appears as an extraordinarily diversified population. Such a chicken population may represent a living gene bank [15]. These results highlight once again the importance of undertaking surveys for domestic species in remote areas when they are large enough to limit genetic drift.

**Figure 2:**
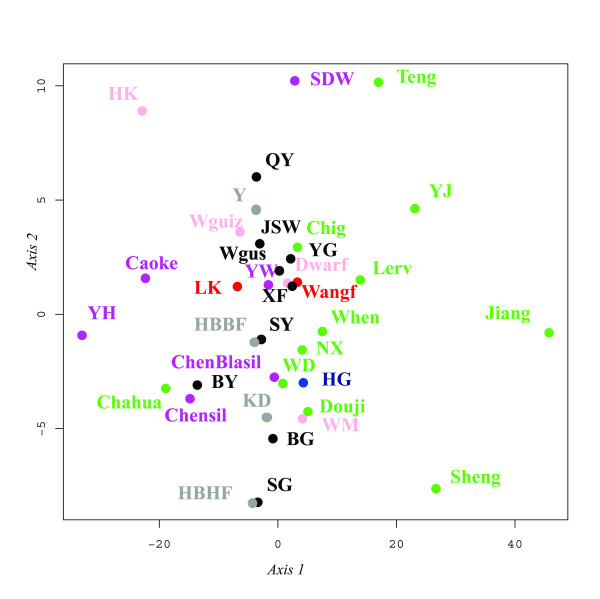
**Multidimensional scaling plot constructed by *K2P *distances among Chinese breeds and the HG population**. Colours correspond to province origin of the breed as in Figure 1.

The degree of population structure was assessed for the Chinese and Vietnamese dataset by calculating the distribution of sequence variations following imposition of two hierarchical groupings (AMOVA analysis). When the breeds were grouped according to geographic location (provinces), the major part of diversity was present within breeds (80.2%), while only a fraction was diagnostic of the provinces (4.8%). The remaining variation was present between breeds within each of the provinces (14%). Multidimensional scaling was constructed using *K2P *distance. This analysis excluded the four breeds that carried only one haplotype. The *K2P *plot (Figure 2) provided information about diversity at the province levels. Breeds from the same province are represented by the same colour, except for black colour which means that the breed is the only representative of its province. Axis 1 seemed to divide breeds from the Sichuan province on the left from breeds from the Yunnan province on the right. Axis 2 was difficult to interpret. However it can be observed that the lower left square included almost all the breeds showing a silky plumage phenotype. The HG population was more similar to the Yunnan breeds which originate in the north Yunnan and the bordering area with the Guizhou province. This area is indeed inhabited by the same ethnic groups as those inhabiting the Ha Giang province. Therefore the HG chicken population is still reflecting the migration history of these ethnic groups [24]. The HG population is also not so far from silky breeds such as the Chengdu Black Silky. The HG population is as yet the only Vietnamese population that can harbour a silky plumage phenotype (Vo Van Su, personal communication). The current HG population is the result of ancestral admixture during the long migration across southern China [24] followed by recent admixture with wild and domestic chickens in the Ha Giang province leading to its high genetic diversity [15]. The absence of the E haplogroup may indicate two important facts. The first one being that no ancestral migration from the Indian sub-continent occurred; indeed all human migrations recorded in this area were from the North to the South [25]. Secondly, as observed during surveys (see [15] for more details), European exotic breeds (commercial lines) have not been introduced in recent decades.

### Geographical pattern and wild admixture within the province

Commune average pairwise differences were only significant for three pairwise commune comparisons: communes 65 and 40; communes 65 and 20; and communes 88 and 7. Two major haplotypes B1 and A1 occurred at frequencies 33% and 11% respectively overall in the HG population (Figure 2). The distribution of the five haplogroups among communes was not significantly variable (Chi^2 ^test; *P *= 0.251). In other words, no geographic distribution pattern of haplogroups was observed in the Ha Giang province. This result, in agreement with nuclear data [15], indicates that there was no substructuring of the HG population.

Regarding the global composition of the mtDNA diversity per population, the HG population is the only one to show the more similar distribution pattern as compared to *G. gallus spadiceus *(percentage of B higher than A, and presence of F, Table 2). This was consistent with the presence of an original domestication centre within the natural distribution range of *G. gallus spadicieus *which ranged from Myanmar to the Chinese Yunnan Province ([26,27]).

In a previous analysis of nuclear diversity [15], four communes (7, 40, 65 and 88) appeared to be highly admixed with Red junglefowl. Grouping these admixed communes, 20 haplotypes were observed from which 8 were also found in the 13 haplotypes observed in the group of non-admixed communes (i.e. 1, 2, 20, 48, 61, and 103). Haplotype diversity was estimated to be 0.866 and 0.861 and nucleotide diversity was 0.129 and 0.134 respectively for admixed and non-admixed. No differences in haplogroup distribution (Chi^2 ^test; *P *= 0.451) between admixed vs. non-admixed were observed. Even if some farmers admit catching eggs in the forest of these admixed communes, the occurrence of such a practice may be lower than hybridisation between wild cocks and domestic hens. This implies that hybrid offspring of crosses between domestic cocks and junglefowl hens are uncommon or rarely back-crossed into the domestic population while the hybrid offspring of domestic females and wild males could be more easily integrated into the domestic population. Thus, such communes can not be discriminated using mtDNA but they can be discriminated with the use of microsatellites which take into account male as well as female mediated gene flows. Examination of wild sympatric populations from the Ha Giang province would be necessary to ascertain the presence or absence of mtDNA gene flow from wild to domestic populations.

### Phylogeographical Asian pattern of mtDNA haplogroups

**Figure 3:**
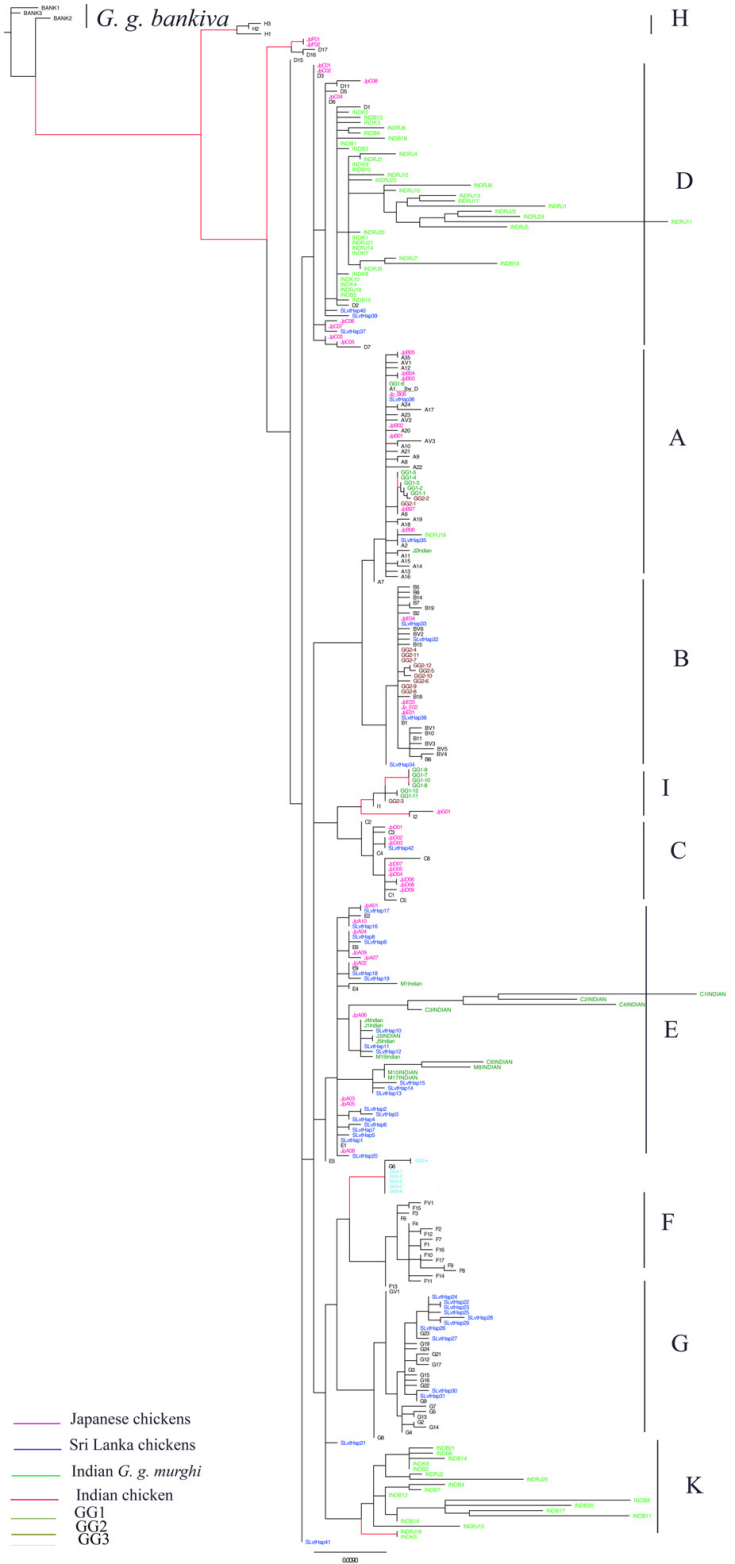
**The PhyML tree of all Asian breeds published data**. Red branch indicate bootstrap values higher than 80%.

**Figure 4:**
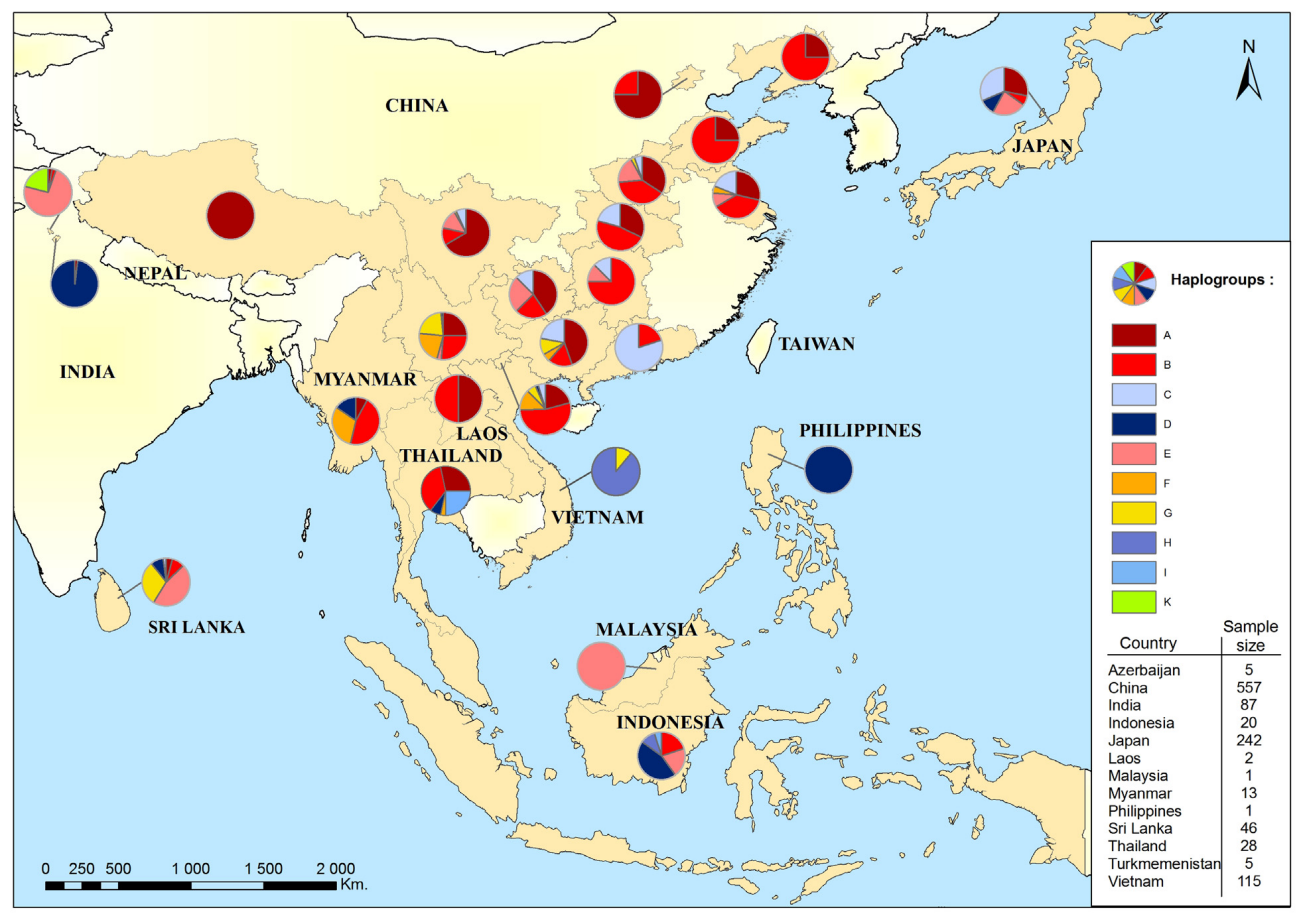
**Distribution and proportions of haplogroups in Asian domestic and wild chickens**. Ha Giang province is in blue.

We combined all sequences published by Oka *et al*. ([9]; Japanese domestic chickens); Kanginakudru *et al*. ([11]; Indian domestic chickens and *G. gallus murghi*) and Silva *et al*. ([10]; Sri Lankan domestic chickens) with haplotypes of this study and from those of Liu *et al*. [7]. We found that the best evolution model fitting the data was HKY+ I (0.711) +G (0.598). When comparing our results to the results obtained by Liu *et al*. [7], we found that D formed the first group comprising haplotypes D1 and D2 and some sequences from wild *G. g. murghi*, and grouped in a second time the remaining D haplotypes with Japanese and Sri Lankan sequences (Figure 3). An important point highlighted by this tree was the subdivision into 2 different clusters of the *G. g. murghi *population. As we showed previously, a first group of sequences fell with D haplotypes. The second group of sequences from *G. g. murghi *formed a cluster apart that we will call K. This group was not clustering with any haplotypes previously described by Liu *et al*. [7]. Moreover, this cluster grouped with the supercluster formed by haplogroups A, B, F, G and E. According to [7], 45% of Indian chickens belong to type D; and 55% belong to haplogroup E which also represented 94% of Indian domestic chickens studied by Kanginakudru *et al*. [11]. Haplogroup E is mainly found in Indian breeds and also in Near Eastern and European breeds [7]. Assuming that the small proportion found in Southeastern Asian breeds (Figure 4) resulted from recent introgression of exotic breeds, we may suggest that this haplogroup originated in the Indian subcontinent. It is interesting to note that haplogroup E was not found in the *G. g. murghi *[11], but only in one other sample of *G. g. gallus *[7]. Therefore two assumptions can be made from these data: (1) the wild population carrying haplogroup E has not been sampled yet, and sampling effort within the natural distribution range of *G. g. murghi *is needed; (2) this haplogroup belongs to an ancestral population from the Indian subcontinent that is now extinct. Furthermore, Haplogroup C has mainly been found in Japanese breeds that originate from commercial exchanges with Korea, Taiwan or the eastern provinces of China [9]. This haplogroup is also frequently present in breeds from the Chinese Guangdong province (Figure 4; Huxu breed: 100% and Qinyuan breed: 67%). Therefore, the area distribution of haplogroup C in the wild population could be the Chinese provinces bordering the South China Sea. Haplogroup A was rather found in Northern Asian breeds while haplogroup F and G were found in the northern part of Southeast Asia. By contrast, Haplogroup B encompassed North and Southeast Asia (Figure 4). Thus, despite human migration and commercial exchanges, we could observe a geographical pattern of haplogroup distribution.

## Conclusions

The Vietnamese HG chicken showed high genetic diversity at both the nuclear and the mitochondrial level when compared to other local breeds. This genetic diversity is the result of many factors. One of the most important is the geographical position within a domestication centre area. Furthermore, we should not underestimate the impact of human and livestock migrations. This genetic diversity has been moreover supplied by the continuous current gene flow between domestic and wild population as shown with nuclear DNA [15]. Hence, the Ha Giang chicken represents a highly valuable genetic resource in constant co-evolution with its environment. The extensive contribution of wild ancestors is a source of increased genetic diversity for farm animals which may acquire the potential for adaptation to environmental changes. All this reinforces the need to maintain such a population.

However, the widespread occurrence of free-ranging livestock is raising fear that introgressive hybridisation with wild populations would lead to a loss of biodiversity in the wild populations that could decrease their fitness. Chazara *et al*. [28] suggested that introgression of domesticated Japanese quail genes in wild Common quail populations might affect the phenotypic expression of functional traits, such as body size, feather colour, sexual calls and migratory behaviour. Continuous contacts between wild and domestic animal also favoured disease exchange. Smith *et al*. [29] showed that virus from the H2N2/1957 and H3N2/1968 pandemics seemed to have originated from avian hosts, probably in Asia. Wild relatives may represent a host population where the virus can evolve and where control measures such as vaccination are not possible to use. Identifying and quantifying such interactions may allow inferring the importance of the wild compartment in the epidemiology of viral infections.

## Authors' contributions

All authors read and approved the final manuscript. NVT carried out sample collection; CB carried out sample collection, sequencing, the computational analysis and prepared the manuscript; GM created the distribution maps, XR and JRM participated in the computational analysis and preparation of the manuscript; BB and NB participated in the sequencing, MTB contributed to the revision of the manuscript; EV participated in the design of the study and the revision of the manuscript; VCC participated in the coordination of the study; JCM participated in the design, coordination of the study, and revision of the manuscript.

## Supplementary Material

Additional file 1 **DOC Samples information**. Summary of breeds used, their origin, number of individuals and GenBank accession numbers. Click here for file
