# Supplementary material for: Vietnamese chickens: a gate towards Asian genetic diversity
Source: BMC Genet. 2010 Jun 18;11:53. doi: 10.1186/1471-2156-11-53 (PMC2897773; doi:10.1186/1471-2156-11-53)
Supplement: Additional file 1 — DOC Samples information. Summary of breeds used, their origin, number of individuals and GenBank accession numbers. [file 1471-2156-11-53-S1.DOC]

**Additional file 1. Samples information.**

| Populations | Province | Country | N ind. | Accession Numbers |
| --- | --- | --- | --- | --- |
| Beijing Youkei | Beijing | China | 4 | AY465980-83 |
| Huxu | Guangdong | China | 5 | AF512215-20 |
| Qinyuan blotted | Guangdong | China | 10 | AF512255-60; AY465964-67 |
| Luke | Guanxi | China | 9 | AF512237-45 |
| Wanfeng Wugu | Guanxi | China | 9 | AF512283-91 |
| Dwarf Wugu | Guizhou | China | 10 | AF512117-27 |
| Guizhou Moutain Wugu | Guizhou | China | 10 | AF512128-37 |
| Heikang Layer | Guizhou | China | 5 | AF512210-14 |
| Wumeng Wugu | Guizhou | China | 4 | AY466000-3 |
| Gushi Wugu | Henan | China | 11 | AF512139-50 |
| Henan Cockfight | Henan | China | 4 | AY465968-71 |
| Taihe Silky | Henan | China | 14 | AF512273-82; AY465988-91 |
| Black Silky hubei | Hubei | China | 10 | AF512200-9 |
| Silhy hybrid | Hubei | China | 3 | AF512234-6 |
| Silky hubei | Hubei | China | 11 | AF512189-99 |
| Yunxian Wugu | Hubei | China | 10 | AF512307-16 |
| Xuefeng | Hunan | China | 8 | AF512299-306 |
| Langshan | Jiangsu | China | 4 | AY466004-7 |
| Silky Jiangsu | Jiangsu | China | 13 | AF512221-33 |
| Yugan wugu | Jiangxi | China | 4 | AY465992-5 |
| Bigbone | Liaoning | China | 4 | AY465976-9 |
| Souguang | Shangdong | China | 4 | AY465984-87 |
| Caoke | Sichuan | China | 16 | AF12091-106 |
| Chengdu Black Silky | Sichuan | China | 9 | AF512267-75 |
| Chengdu Silky | Sichuan | China | 6 | AF512060, 62-66 |
| SichuanMountain Wugu | Sichuan | China | 11 | AF512262-72 |
| Ya'an Non Wugu | Sichuan | China | 7 | AF512317-23 |
| Ya'an Wugu | Sichuan | China | 10 | AF512328-37 |
| Tibetan | Tibetan | China | 4 | AY465960-63 |
| Chahua | Yunnan | China | 16 | AF512076-90; AY465972-75 |
| Chigulu | Yunnan | China | 19 | AY392172-90 |
| Douji | Yunnan | China | 9 | AF512108-116 |
| Jiangbian | Yunnan | China | 28 | AY392205-32 |
| Lv'erwu | Yunnan | China | 29 | AY392233-61 |
| Nixi | Yunnan | China | 9 | AF512246-54 |
| Shenggou | Yunnan | China | 3 | AF512057-9 |
| Tenchongxue | Yunnan | China | 45 | AY392296-340 |
| Whenshanshandi | Yunnan | China | 35 | AY392342-76 |
| Wuding | Yunnan | China | 7 | AF512292-98 |
| Yanjing Wugu | Yunnan | China | 8 | AF512324-27; AY465996-99 |
| Ha Giang | Ha Giang | Vietnam | 106 | This study : HM462082-187 |
| Sri Lanka domestic |  | Sri Lanka | 42 | EU199906-47 |
| Japan |  | Japan | 42 | AB268506-45; AB294232-3 |
| Indian domestic |  | India | 16 | EU847801-16 |
| *G. g. murghi* |  | India | 56 | EU847745-800 |
| *G. g. spadiceus* |  | Myanmmar, Laos, Thailand, China (Yunnan) | 36 | AB009442-3;AF512154-88 |
| *G. g. spadiceus* |  | Thailand | 12 | This study : HM462196-207 |
| *G. g. jabouillei* |  | China (Yunnan) | 2 | AF512151-52 |
| *G. g. gallus* |  | Thailand, Philippines, Indonesia, Vietnam and unknow | 22 | D82900-8;AB007720-23; AB007725; AB007752-3; AB007756-7; AB009432-43 |
| *G. g. gallus* |  | Thailand | 12 | This study : HM462208-17 |
| *G. g. gallus* |  | Vietnam | 6 | This study : HM462188-95 |
| *G. g. bankiva* |  | Indonesia | 3 | AB007718; AB009430-1 |
